# Extraction and Purification of R-Phycoerythrin Alpha Subunit from the Marine Red Algae *Pyropia Yezoensis* and Its Biological Activities

**DOI:** 10.3390/molecules26216479

**Published:** 2021-10-27

**Authors:** Selvakumari Ulagesan, Taek-Jeong Nam, Youn-Hee Choi

**Affiliations:** 1 Department of Marine Bio-Materials & Aquaculture, Pukyong National University, Nam-gu, Busan 48513, Korea; selva@pknu.ac.kr; 2 Institute of Fisheries Sciences, Pukyong National University, Gijang-gun, Busan 46041, Korea; namtj@pknu.ac.kr

**Keywords:** *Pyropia yezoensis*, marine algae, phycoerythrin, antioxidant activity, cytotoxicity, anticancer activity

## Abstract

Phycoerythrin is a major light-harvesting pigment of red algae and cyanobacteria that is widely used as a fluorescent probe or as a colorant in the food and cosmetic industries. In this study, phycoerythrin was extracted from the red algae *Pyropia yezoensis* and purified by ammonium sulfate precipitation and various chromatography methods. The purified phycoerythrin was analyzed by UV-visible and fluorescence spectroscopy. The isolated pigment had the typical spectrum of R-phycoerythrin, with a trimmer state with absorbance maxima at 497, 536, and 565 nm. It was further purified and identified by LC-MS/MS and Mascot search. It showed a 100% sequence similarity with the R-phycoerythrin alpha subunit of *Pyropia yezoensis*. The molecular mass was 17.97 kDa. The antioxidant activity of the purified R-phycoerythrin alpha subunit was analyzed. It showed significant antioxidant activity in ABTS and FRAP assays and had significant cytotoxicity against HepG2 cells.

## 1. Introduction

Hepatocellular carcinoma and lung carcinoma are the most prevalent forms of cancers reported globally [1]. Although chemotherapeutic drugs can be pretty effective in treating hepatocellular carcinoma and lung carcinoma, these agents do not differentiate normal healthy cells from the cancerous cell [2]. Free radicals and natural anticancer drugs can directly induce the formation of cancer cells in the human body, thus chemopreventive agents have gained popularity in cancer treatment. Hence, radical scavenging compounds, such as natural pigments from marine algae, can indirectly reduce cancer formation in the human body. Marine algae-derived natural pigments are known to be important free radical scavengers and antioxidants for the prevention of oxidative damage, which is an important contributor in carcinogenesis [3]. Most natural anticancer compounds can manipulate the growth of cancer cells with negligible or minor side effects [4].

Therefore, the identification of novel effective cancer chemopreventive agents and therapeutic agents has become a crucial worldwide strategy in cancer prevention and treatment (mainly to overcome drug resistance and reduce toxicity to the host), respectively. Given this, phycobiliproteins from red macroalgal species have an important role as anticancer agents due to their high efficiency and low toxicity. They can enhance the activity of conventional anticancer medicines, decreasing their side impacts [5]. The development of a suitable antioxidant molecule is gaining more importance today as it plays a key role in preventing or delaying hepatotoxicity, heart diseases, and cancer. The food and pharmaceutical industries use several synthetic commercial antioxidants, such as butylated hydroxyanisole (BHA), butylated hydroxytoluene (BHT), tert-butylhydroquinone (TBHQ), propyl gallate (PG), and tocopherol, to retard the oxidation and peroxidation process as well as a food additive [6,7]. Previous studies suggested that the use of these synthetic antioxidants could be toxic and lead to potential health hazards (i.e., destroying the liver and causing cancer). The use of artificial antioxidants has been reduced due to their carcinogenic nature. So, there is a crucial need to substitute them with new harmless natural antioxidants. Hence, the safe replacement of synthetic antioxidants with natural antioxidants could be beneficial due to health implications and functionality in the food and pharmaceutical industry. Additionally, there is considerable interest in the food and pharmaceutical industry for the development of antioxidants from natural sources, such as marine flora and fauna. Among marine resources, marine algae represent one of the richest sources of natural antioxidants [8].

Artificial dyes have been approved for their use in the food industry; however, some of them have been reported to be neurotoxic, mutagenic, and genotoxic (lemon-yellow tartrazine), damaging the liver and kidney (brilliant blue), and triggering biochemical changes and cancer in the thyroid gland (cherry-red erythrosine) [9,10,11]. The demand for natural dyes in the food industry has grown in recent years due to the toxicity of artificial colorants [12]. There is great interest in the identification of non-harmful alternative pigments that can scavenge hydroxyl ions to avoid lipoperoxidation [13]. Natural dyes can be considered renewable and sustainable bioresources with minimal environmental impact [14]. These eco-friendly, presumably mostly non-toxic, natural colorants could have applications in other industrial sectors like the cosmetics or pharmacological industries [15,16,17,18]. Based on the literature review, the natural pigments derived from marine algae are the best replacement for artificial dye used in the food and pharmaceutical industry.

Marine algae have served as an essential source of bioactive natural products. Moreover, many metabolites isolated from marine algae have been shown to possess biological activities and potential health benefits. They are also rich in natural pigments, besides their role in photosynthetic and pigmentation effects [19]. The pigments found in marine algae are phycobiliproteins, which are water-soluble fluorescent proteins [20]. Phycobiliproteins are classified into four main groups based on their light absorption properties and the types of bilin: phycoerythrins (λ_max_ 540–570 nm), phycocyanins (λ_max_ 610–620 nm), phycoerythrocyanins (λ_max_ 560–600 nm), and allophycocyanins (λ_max_ 650–655 nm) [20,21]. Phycoerythrin is a major water-soluble light-harvesting pigment in red algae (Cyanobacteria), Rhodophyta, Glaucocystophyta, and Cryptophyta. Phycoerythrins are subdivided into three main types: B-phycoerythrin (B-PE; λ_max_ = 565 and 546 nm and a shoulder at 499 nm), C-phycoerythrin (C-PE; λ_max_ = 565 nm), and R-phycoerythrin (R-PE; λ_max_ = 565 and 498 nm, and a shoulder at 540 nm) [20,22,23]. R-phycoerythrin is the most abundant phycobiliprotein found in red algae [24] and is commonly composed of 6α, 6β, and 1γ subunits [25].

Red macroalgae can grow in deep water due to their high content of phycoerythrins, which efficiently absorb light at 450–570 nm [26]. The marine red algae *Pyropia yezoensis* belongs to the class Bangiophyceae in Rhodophyta and is cultivated for human consumption in Korea, China, and Japan [27]. The protein content of dried Pyropia is 41.4%, nearly three times that of other seaweeds [28]. Antioxidants from seaweed have attracted interest in pharmaceutical production since these compounds prevent or retard the adverse effects of free radicals. Phycobiliproteins are hydrophilic and have in vitro and in vivo antioxidant activity toward free radicals and selenium [29]. Phycobiliproteins have essential roles as anticancer agents due to their high efficiency and low toxicity. They can enhance conventional anticancer medicines’ activity, decrease their side effects, and act as photosensitizers to treat affected cells [5]. This study highlighted the antioxidant activity and cytotoxicity effects of phycobiliprotein, mainly phycoerythrin isolated and purified from *Pyropia yezoensis*, against HepG2 hepatocellular carcinoma cells.

## 2. Results and Discussion

### 2.1. Isolation and Purification of R-Phycoerythrin Alpha Subunit from Pyropia Yezoensis

**Figure 1:**
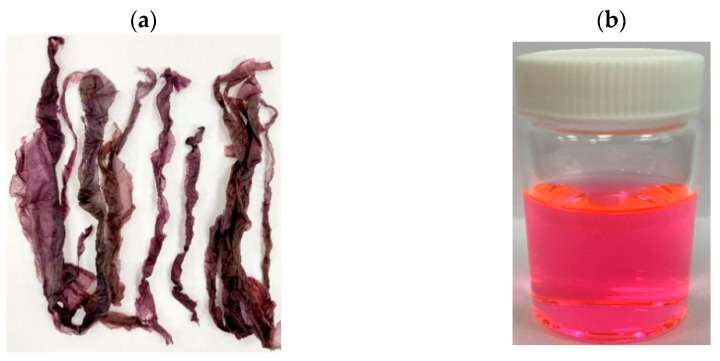
(**a**) The marine algae *Pyropia yezoensis*; (**b**) purified R-phycoerythrin.

The crude extract of *Pyropia yezoensis* was extracted and saturated with 80% ammonium sulfate. It was desalted using a 2 kDa dialysis membrane. The crude protein concentration was measured by the BCA assay method [30]. It was about 10% (100 mg/g) of the total biomass. The crude protein was separated into fractions by FPLC. The peak-containing fractions were combined (Figure 1) and analyzed with UV-visible and fluorescence spectrometry. R-phycoerythrin (R-PE) is an intensely bright phycobiliprotein isolated from red algae that exhibits extremely bright red-orange fluorescence with high quantum yields. It is excited by laser lines from 488 to 561 nm, with absorbance maxima at 496, 546, and 565 nm and a fluorescence emission peak at 578 nm.

**Figure 2:**
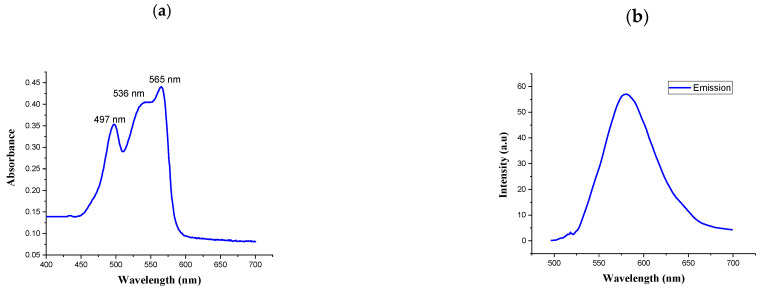
(**a**) Absorption spectrum of R-phycoerythrin; (**b**) fluorescence spectrum of R-phycoerythrin after excitation at 565 nm.

UV-vis spectra and fluorescence spectra were measured to characterize the spectral properties of R-PE. As shown in Figure 2a,b, the absorption spectra of the purified phycoerythrin were determined by UV-visible spectrophotometry at 200–800 nm. The absorption spectra had three peaks: two at 497 and 536 nm and a main peak at 565 nm. The highest peak was measured by fluorescence spectrophotometry. The spectral profile is commonly used to indicate the non-degradation of phycoerythrin [31]. It presented a typical absorption spectrum of R-PE. Similarly, the UV-visible absorption spectrum of the R-phycoerythrin obtained from *B. atropurpurea* showed a peak at 530–570 nm, indicating the presence of R-phycoerythrin [32].

The fluorescence emission spectra represent the functional properties of the isolated R-PE. The fluorescence emissions at 575 nm when it was excited at 495 nm were recorded for R-PE. This result was similar to the published evidence for the fluorescence spectrum of R-PE [33].

**Figure 3:**
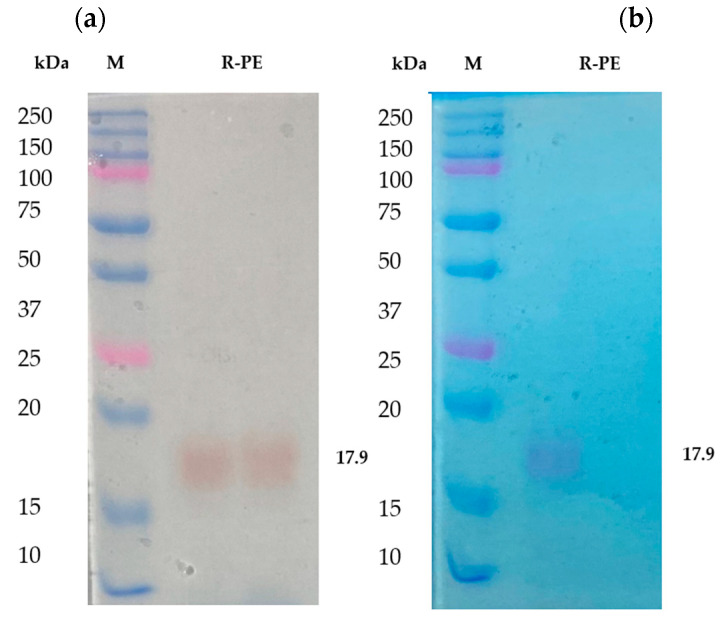
The purified R-phycoerythrin alpha subunit was analyzed in 12.5% SDS-PAGE. (**a**) Unstained polyacrylamide gel with M: molecular weight marker, R-PE-purified phycoerythrin alpha subunit from *Pyropia yezoensis.* (**b**) Polyacrylamide gel stained with Coomassie brilliant blue R-250, 10 μg of protein sample were loaded in each well M: molecular weight marker; R-PE-purified phycoerythrin alpha subunit from *Pyropia yezoensis.*

From the results, it has been noted that the separated fractions contain R-phycoerythrin in a trimmer state. It was further purified by RP-HPLC and identified as a single band in SDS-PAGE. It was found that the molecular weight was approximately 17 to 18 kDa (Figure 3a), which was identified as R-phycoerythrin alpha subunit by LC-MS/MS. The purified phycoerythrin alpha subunit concentration was measured by BCA assay. It was about 1.5 mg/g of the dry weight of the total biomass. Other researchers have reported that the phycoerythrin (PE) concentration of purified red algae, *Portieria hornemannii, Gracilaria corticata*, and *Gelidiella acerosa* ranged from 0.39 to 1.23 mg/g of the dry weight concentration [34,35]. This shows that the concentration of phycoerythrin in *Pyropia yezoensis* is more than the average total content of R-PE than other red algae species.

R-phycoerythrin alpha subunit was extracted and purified from the marine algae *Pyropia yezoensis*. The purity index of the phycoerythrin was approximately 5.4. The purity of the protein was determined by calculating the ratio between the OD of all protein at 280 nm and that of the specific protein, in this case, 565 nm for phycoerythrin. The R-PE extracted from *P. yezoensis* had a purity of 2.8 [36]. The purity index of R-PE obtained from *Grateloupia turuturu* was 1.07 [37]. A purity index above two is considered suitable for the food and cosmetic industries [25].

Previous studies also suggested that the protein nature, unique color, and fluorescence efficiency of R-PE extend its uses in the food and cosmetic industry as a natural dye and as a marker in gel-electrophoresis and isoelectrofocusing [38] based on its purity [20]. SDS-PAGE is helpful in the determination of the molecular weight of R-phycoerythrin. In the present study, the purified R-PE alpha subunit was analyzed in 12.5% SDS PAGE [39] and stained with Coomassie brilliant blue R-250. It was compared with unstained gel. The color and molecular weight are shown in Figure 3b. The aboveaverage R-PE content and higher R-PE purity index of *Pyropia yezoensis* make these algae the best source of its R-PE and antioxidant properties.

### 2.2. MS2 In-Gel Digestion and Protein ID by LC-MS/MS

**Figure 4:**
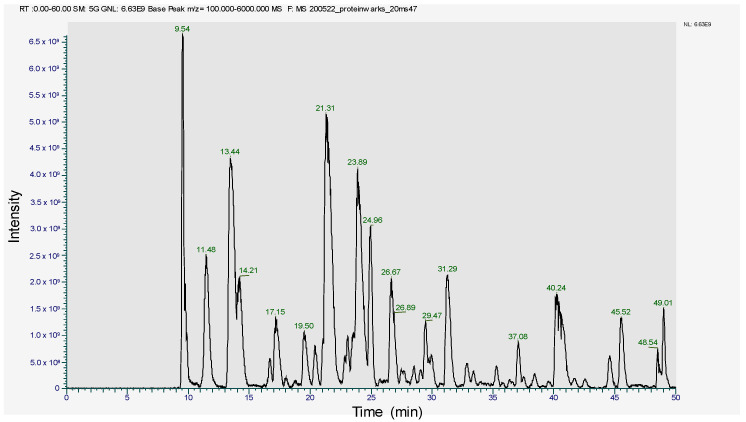
Peptide profile image of R-phycoerythrin alpha subunit.

The SDS-PAGE band of 17~18 kDa was subjected to trypsin digestion and analyzed in LC-MS/MS. The peptide profile was obtained and is presented in Figure 4. The subsequent LC-MS/MS data on Mascot search identified 34 peptides (Table 1). Mascot analysis revealed that these peptides showed a 100% sequence similarity with the R-Phycoerythrin alpha subunit of *Pyropia yezoensis* compared to the other proteins of this species, such as Phycoerythrin beta subunit (77.4%), C-phycocyanin beta chain (84.9%), Allophycocyanin alpha subunit (49.7%), C-phycocyanin alpha chain (24.1%), and Ribosomal protein L12 (15.5%). However, the molecular weight of R-phycoerythrin alpha subunit (17972 Da) is in agreement with the molecular weight (17.9 kDa) observed from the SDS-PAGE (Figure 3, Table 2). The R-phycoerythrin is considered a multimeric protein, including 17.9 kDa alpha subunits. These results are consistent with the previously published research from marine red algae, as the molecular weight range of R-phycoerythrin alpha subunit falls between 16 and 20 kDa. [40]. This study elucidates the usefulness of various bioanalytical techniques, including identifying and characterizing novel protein isolates from marine macroalgae.

### 2.3. Antioxidant Activity

Natural antioxidants, found in marine algae, are important bioactive compounds that play an essential role against various diseases and aging processes by protecting cells from oxidative damage [3]. In the present study, R-phycoerythrin alpha subunit from the marine algae *Pyropia yezoensis* was extracted and analyzed for its antioxidant properties.

**Figure 5:**
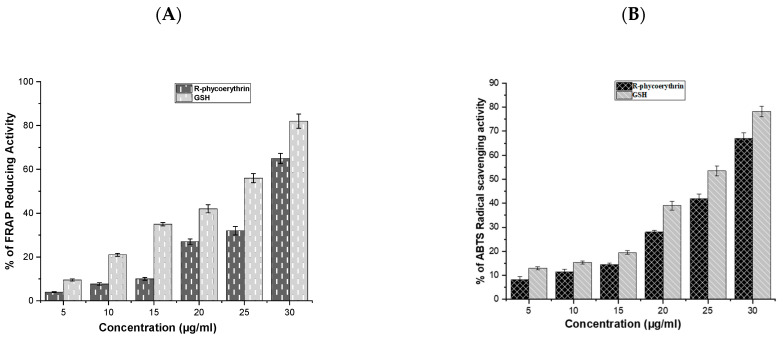
The radical scavenging activity of R-phycoerythrin alpha subunit (**A**) ABTS, (**B**) FRAP assays. R-phycoerythrin alpha subunit exhibited a dose-dependent increase in radical scavenging activity. This demonstrated that the phycoerythrin from *P. yezoensis* is a source of natural antioxidants that have a potential protective role against oxidative stress and biotechnological applications in the food and pharmaceutical industries. The results suggested that R-phycoerythrin alpha subunit is a relatively powerful antioxidant. The assays were done in triplicate and the results are shown as the mean and standard deviation.

The antioxidant activity of the purified phycoerythrin was analyzed using ABTS and FRAP assays and is presented in Figure 5A,B. The antioxidant activity of R-phycoerythrin alpha subunit increased in a dose-dependent manner, which was compared with GSH (glutathione).

Antioxidants may have a positive effect on human health as they can protect the human body against damage by ROS, which attack macromolecules, such as membrane lipids, proteins, and DNA, leading to many health disorders, such as cancer, diabetes mellitus, aging, and neurodegenerative diseases [41,42]. The reducing power was determined based on the ability of antioxidants to reduce Fe^3+^ to Fe^2+^ [43]. Fe^3+^ reduction is often used as an indicator of electron-donating activity, which is an antioxidant reaction. In the present study, the purified R-phycoerythrin alpha subunit shows the reducing power in a dose-dependent manner. Similar results were presented in the previous literature, such as [44], who reported that extracts of *Spirulina platensis* exhibited antioxidant activity (99.5% inhibition of ABTS) at 200 μg/mL. The author of [45] reported that extracts of *Nostoc linckia* (70% inhibition of ABTS radical) at 5 mg/L and ABTS radical scavenging capacity increased with the increasing phycobiliproteins concentration and reached up to 77%, 85%, and 92% at 10, 15, and 20 mg/L, respectively. The authors of [44] reported 73% inhibition in *Oscillatoria* and 76.8% inhibition in *Nostoc muscorum* against standard BHT (85.6% inhibition). The results of the study suggested that the R-phycoerythrin alpha subunit from *Pyropia yezoensis* is a relatively powerful antioxidant.

### 2.4. Cytotoxicity of R-Phycoerythrin Alpha Subunit

**Figure 6:**
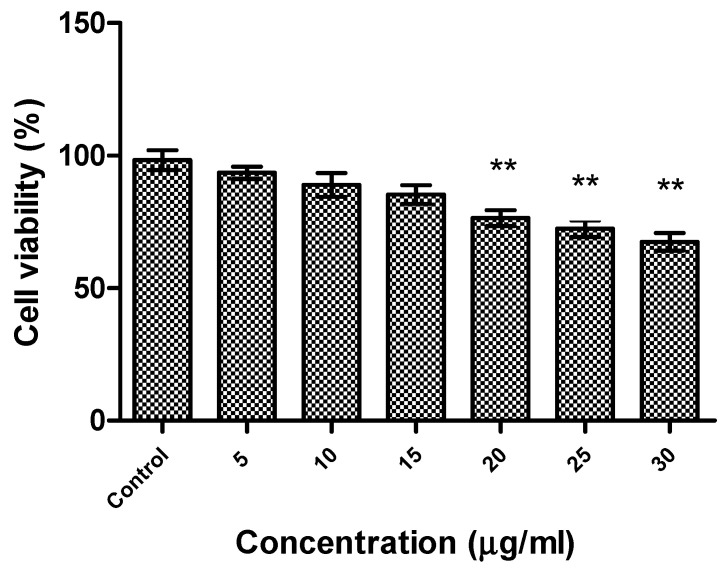
Cytotoxicity of R-phycoerythrin alpha subunit from *Pyropia yezoensis* analyzed in HepG2 cells. One-way ANOVA was performed to understand the significant difference between the control group and the R-PE alpha subunit-treated groups. The R-PE alpha subunit treated groups (20, 25, and 30 µg/mL) showed significance activity, ** indicates (*p* < 0.01).

The effect of R-phycoerythrin alpha subunit on human hepatocellular carcinoma cells (HepG2) was analyzed by in vitro cytotoxicity assay using the MTS reduction method. A cell cytotoxicity assay revealed that R-phycoerythrin alpha subunit extracted from the marine algae *Phyropia yezoensis* served as an anticancer agent in HepG2 cells at 5–30 μg/mL (Figure 6). The cell viability decreased with increasing concentrations of purified R-PE α. It showed significant activity when compared with the control group. Similarly, the anticancer activity of PE from the red algae *Gracilaria cortica* against HepG2 cells was also reported [46]. Shanab et al. [34,47] reported that the R-phycoerythrin from red algae *Portieria hornemannii* showed cytotoxicity against HepG2 cells in a dose-dependent manner. The previous study reported high anticancer activity of PE extracted from Oscillatoria and Nostoc muscorum against HepG2 cells (77.8% and 89.4% inhibition, respectively).

**Figure 7:**
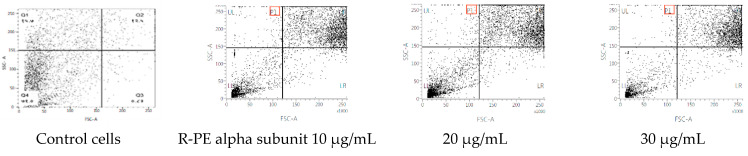
Flow cytometric analyses of R-phycoerythrin alpha subunit-treated cells.

The cycle distribution effect of R-phycoerythrin alpha subunit was evaluated by the fluorescence-activated cell sorting (FACS) flow cytometry method with annexin V/PI staining to clarify the mechanism of R-phycoerythrin alpha subunit induction of death in HepG2 cells after 24 h of exposure and measurement of the fluorescence by flow cytometry. Figure 7 shows each cell subpopulation. These results indicate that the R-phycoerythrin alpha subunit altered the cell membrane integrity by increasing the percentage of PI-positive cells. The R-phycoerythrin alpha subunit-treated HepG2 cells showed a decrease in the G2/M phase compared to the control. However, the percentage of S phase in treated cells was increased. This increment was coupled with an increase in the percentage of cells in the G0/G1 phase. The observed accumulation of cells in the G2/M phase accompanied by cell cycle arrest in the flow cytometry studies indicated that the R-PE alpha subunit protein of *Pyropia yezoensis* induced apoptosis in both cell lines via the G0/G1 and S phases of the cell cycle. Similar results were reported by [34].

## 3. Materials and Methods

### 3.1. Chemicals

The reagents used in this study, including ammonium sulfate, trypsin, 2,2′-azino-bis (3-ethylbenzothiazoline-6-sulphonic acid (ABTS), 2,4,6-tripyridyl-s-triazine (TPTZ), ferric chloride, and glutathione (GSH), were purchased from Sigma-Aldrich. The 2 kDa dialysis membrane was purchased from Spectrum Chemicals (New Brunswick, NJ, USA), and a Dead Cell Apoptosis Kit with Annexin V FITC and PI for flow cytometry was purchased from Thermo Fisher Scientific (Waltham, MA, USA). All reagents and chemicals used in this experiment were of analytical grade. Reactions were performed in deionized water.

### 3.2. Preparation of Pyropia Yezoensis Extract

The marine red algae *P. yezoensis* was obtained from Suhyup (Busan, South Korea), freeze-dried, and ground into a powder. Five grams of the powder were suspended in 100 mL of ultra-pure water in a 500-mL flask; sonicated using the (QSONICA sonicators USA Q500, Newtown, CT, USA) with 5 pulses of 5 s each, giving a pause of 5 s in between each pulse, for 1 h at 400 rpm under ice; and centrifuged at 45,000× *g* for 20 min at 4 °C. The soluble protein was collected in the supernatant and precipitated by 80% ammonium sulfate. Dialysis was performed using a 2 kDa dialysis membrane to remove the ammonium sulfate from the precipitated pellet against distilled water. The soluble protein was collected, freeze-dried, and stored at 4 °C.

### 3.3. Purification

The protein was further purified by fast protein liquid chromatography (AKTA Prime Plus, GE Healthcare, Piscataway, NJ, USA) using a HiPrep Sephacryl S-200 HR 16/60 column (GE Healthcare, Chicago, IL, USA) pre-equilibrated with 50 mM Tris-HCl pH 7.2. In total, 2 mL of protein sample (1 mg/mL) were injected and 80 fractions were collected. The peak-containing fractions were combined together and analyzed with UV-Vis and fluorescence spectrometry. Further, it was purified by reverse-phase high-performance liquid chromatography with a Sep-Pak plus C18 reversed-phase column (Waters, Milford, MA, USA). The bound protein was eluted with reversed-phase buffer A (0.1% [vol/vol]) trifluoroacetic acid) containing 20, 40, 60, or 80% acetonitrile. Following separation, the acetonitrile was evaporated from the protein solution, and the protein content of all fractions was analyzed using SDS-PAGE. The phycoerythrin alpha subunit was identified as a single band corresponding to a molecular weight of 17.9 kDa. The resulting R-phycoerythrin alpha subunit was freeze-dried.

The lyophilized R-phycoerythrin alpha subunit was resuspended in ultra-pure water at 1 mg/mL by gentle agitation for 15–30 min at room temperature. The completely dissolved R-phycoerythrin alpha subunit was stored at 4 °C. The protein concentration was determined using bovine serum albumin as the protein standard by the bicinchoninic acid assay. The molecular weight was determined by 12.5% SDS-PAGE.

### 3.4. MS2 In-Gel Digestion and Protein Identification by Automatic LC-MS/MS

Proteins were subjected to in-gel trypsin digestion [48]. The excised gel band was destained with 100 μL of destaining solution (50% ACN/50 mM ABC) and shaken for 5 min. After removing the solution, the gel spots were incubated with 200 mM ammonium bicarbonate for 20 min. The gel pieces were dehydrated with 100 μL of acetonitrile and dried in a vacuum centrifuge. The procedure was repeated three times. The dried gel pieces were rehydrated with 20 μL of 50 mM ammonium bicarbonate containing 0.2 μg of modified trypsin (Promega, Madison, WI, USA) for 45 min on ice. After removing the solution, 70 μL of 50 mM ammonium bicarbonate were added. The protein was digested overnight at 37 °C. The peptide solution was desalted using a homemade C18 nano column. The protein was identified by LC-MS/MS ion searches using Mascot^®^ ver. 2.4.1 (Matrix Science, London, UK).

For protein analysis, ultimate 3000 (Thermo) LC coupled with Q exactive plus MS was used. Digested protein samples were passed through a pre-made analytical column (with a pore size of 75 μm i.d., 15 cm, C18, 100A, 2 μm). Then, 0.1% formic acid in distilled water and 0.1% formic acid in 90% acetonitrile were used as mobile phases A and B, respectively. The flow rate maintained in the separation column was 300 nL/min. The mobile phase gradient at the nano pump was maintained as follows: 0 min, 5% B; 5 min, 5% B; 12 min, 15% B; 45 min, 60% B; 45 min, 60% B; 47.5 min, 95% B; 52.5 min, 95% B; 55 min, 5% B; 60 min, 5% B. All MS data were acquired using positive polarity with the ESI-TRAP instrument. The acquired raw data of MS were converted to a mascot generic format file (.mgf) using Mass Hunter software and searched against a protein database using a Mascot (version 2.4.1, Boston, MA, USA) search engine. For Mascot searches, carbamidomethylated cysteines (C) were set as fixed modifications, and oxidation of methionine (M) was set as variable modifications. The peptide mass tolerance and fragment mass tolerance were 10 ppm and 0.8 Da, respectively.

### 3.5. Antioxidant Activity

#### 3.5.1. ABTS Assay

The method used was as described by [49]. ABTS assays were performed with R-phycoerythrin alpha subunit. Stock solutions of 7 mM ABTS and 2.4 mM potassium persulfate were prepared separately, mixed in equal volumes (1:1), and allowed to react for 12–16 h in the dark. After incubation, the mixed ABTS solution was diluted with methanol to obtain a solution with an absorbance of 0.700 ± 0.03 at 734 nm. R-phycoerythrin alpha subunit (100 μL of 5, 10, 15, 20, 25, and 30 μg/mL) was then added to 2 mL of the ABTS solution, and the absorbance of the mixture was recorded at 734 nm after 1 min of incubation at room temperature. GSH was used as a reference standard.

#### 3.5.2. Ferric-Reducing Antioxidant Power (FRAP) Assay

The FRAP was measured following [50]. FRAP assays were performed with R-phycoerythrin alpha subunit. Stock solutions of 300 mM acetate buffer (3.1g C_2_H_3_NaO_2_ × 3H_2_O and 16 mL C_2_H_4_O_2_, pH 3.6), 10 mM TPTZ (2,4,6-tripyridyl-s-triazine) in 40 mM HCl, and 20 mM FeCl_3_ × 6H_2_O were prepared. Fresh working solutions were prepared by mixing (10:1:1) 25 mL of acetate buffer, 2.5 mL of TPTZ solution, and 2.5 mL of FeCl_3_ × 6H_2_O solution and warming the mixture at 37 °C before use. R-phycoerythrin alpha subunit (40 μL of 5, 10, 15, 20, 25, and 30 μg/mL) was then added to 200 μL of the FRAP solution, and the absorbance of the resulting mixture was recorded at 593 nm after 10 min of incubation at room temperature. GSH was used as a reference standard.

### 3.6. Cell Culture

The human hepatoma carcinoma cell line HepG2 was obtained from the American Type Culture Collection (Manassas, VA, USA). Cells were maintained in a humidified 5% CO_2_ incubator at 37 °C in Dulbecco’s modified Eagle’s medium (DMEM; Gibco, Thermo Fisher Scientific, Waltham, MA, USA) supplemented with 10% fetal bovine serum (FBS; Gibco), 100 U/mL penicillin (Gibco), and 100 µg/mL streptomycin (Gibco). Cultures were viewed under an inverted microscope to evaluate confluence and the absence of bacterial and fungal contaminants.

### 3.7. MTS Assay

Cell viability was analyzed using the CellTiter 96 aqueous non-radioactive cell proliferation assay (Promega), which is based on the formation of a formazan product from 3-4,5-dimethylthiazol-2-yl)-5-(3-carboxymethoxyphenyl)-2-(4-sulfonyl)-2H-tetrazolium (MTS). Briefly, HepG2 cells (5 × 10^4^/well) were seeded into 96-well plates in 100 μL of DMEM supplemented with 10% FBS and incubated at 37 °C for 24 h to allow surface attachment. The medium was then removed and replaced with increasing concentrations (5 to 30 µg/mL) of R-phycoerythrin alpha subunit suspended in a serum-free medium (minimum of three wells seeded for each concentration). The R-phycoerythrin alpha subunit-treated cells were incubated for 24 h at 37 °C. MTS solution (10 μL) was added to the cell suspensions and incubated at 37 °C for 30 min, after which the absorbance at 490 nm was measured using a Gen5 microplate reader (Bio-Tek, Houston, TX, USA) [51]. Experiments were performed in triplicate.

### 3.8. Flow Cytometry Analyses

Apoptotic cells were identified with a dual staining method. HepG2 cells (1 × 10^6^/well) were cultured in 6-well plates and incubated for 24 h. After incubation, the medium was removed and replaced with a medium containing the IC50 concentration of R-phycoerythrin alpha subunit-untreated control cells as a negative control. After 24 h, the cells were washed three times with phosphate-buffered saline and harvested by spreading 1 mL of trypsin over the surface of the tissue culture flask and incubated for 3–5 min. Following incubation, the detached cells were rinsed with medium, transferred to a centrifuge tube, and centrifuged at 1000 rpm for 5 min. The supernatant was discarded, and the pellet was resuspended in 500 µL of 1× binding buffer. The cells were stained with 5 µL of annexin V-FITC conjugate and 10 µL of propidium iodide (PI) solution in the dark at 30 °C for 15 min, and then analyzed by flow cytometry (CytoFLEX, Beckman Coulter, Indianapolis, IN, USA) [52].

### 3.9. Statistical Analysis

All statistical analyses were performed in OriginPro v8 (OriginLab, Northampton, MA, USA), and the values are expressed as the mean ± standard deviation. Statistical significance was calculated using one-way ANOVA.

## 4. Conclusions

The R-phycoerythrin alpha subunit was purified from *Pyropia yezoensis* by ammonium sulfate precipitation and fast protein liquid chromatography. It was identified by Mascot search analysis. The molecular weight of the isolated R-phycoerythrin alpha subunit was 17.97 kDa and the purity index was 5.4. The R-phycoerythrin alpha subunit showed excellent antioxidant activity in ABTS and FRAP assays and showed significant cytotoxicity against HepG2 cells. This study concludes that the R-phycoerythrin alpha subunit from *Pyropia yezoensis* is a promising natural antioxidant and anticancer agent for the food and pharmaceutical industries. Further research evaluating the in vivo activity of R-phycoerythrin alpha subunit from *Pyropia yezoensis* will be useful in the food and pharmaceutical industries.

## Figures and Tables

**Table 1:** Identified peptide list of R-phycoerythrin alpha subunit.

Protein	Sl.No	Identified Peptides	Enzyme Used	Charge State	Observe Mass (Da)	Calculated Mass (Da)	Software Used For Peptide Identification
R-Phycoerythrin alpha subunit	1	VNKCYR	Trypsin	2	838.4112	838.4119	Mascot
	2	DRLCVPR	Trypsin	2	914.4727	914.4756	Mascot
	3	DVDHYMR	Trypsin	2	934.3965	934.3967	Mascot
	4	EAGDACFAK	Trypsin	2	967.4037	967.4069	Mascot
	5	AAARLEAAEK	Trypsin	2	1028.558	1028.561	Mascot
	6	LASNHEAVVK	Trypsin	3	1066.574	1066.577	Mascot
	7	NPGEAGDSQEK	Trypsin	2	1130.484	1130.484	Mascot
	8	CYRDVDHYMR	Trypsin	4	1413.589	1413.592	Mascot
	9	SVITTTISAADAAGR	Trypsin	2	1432.753	1432.752	Mascot
	10	NPGEAGDSQEKVNK	Trypsin	3	1471.689	1471.69	Mascot
	11	EAGDACFAKYSYLK	Trypsin	2	1621.732	1621.745	Mascot
	12	MKSVITTTISAADAAGR	Trypsin	3	1691.889	1691.888	Mascot
	13	LEAAEKLASNHEAVVK	Trypsin	3	1707.914	1707.916	Mascot
	14	FPSSSDLESVQGNIQR	Trypsin	3	1762.848	1762.849	Mascot
	15	VNKCYRDVDHYMR	Trypsin	5	1770.792	1770.793	Mascot
	16	Y*SYLKNPGEAGDSQEK	Trypsin	2	1784.818	1784.822	Mascot
	17	TLNLPTSAYVASFAFAR	Trypsin	3	1827.945	1827.952	Mascot
	18	NPGEAGDSQEKVNKCYR	Trypsin	4	1950.876	1950.885	Mascot
	19	LASNHEAVVKEAGDACFAK	Trypsin	3	2015.969	2015.974	Mascot
	20	AAARLEAAEKLASNHEAVVK	Trypsin	4	2077.131	2077.128	Mascot
	21	TLNLPTSAYVASFAFARDR	Trypsin	3	2099.083	2099.08	Mascot
	22	YSYLKNPGEAGDSQEKVNK	Trypsin	3	2126.024	2126.028	Mascot
	23	EVYRTLNLPTSAYVASFAFAR	Trypsin	4	2375.234	2375.227	Mascot
	24	LVNYCLVVGGTGPVDEWGIAGAR	Trypsin	3	2402.2	2402.205	Mascot
	25	DMSAQAGVEYAGNLDYIINSLC	Trypsin	2	2403.068	2403.072	Mascot
	26	LEAAEKLASNHEAVVKEAGDACFAK	Trypsin	3	2657.287	2657.312	Mascot
	27	TLNLPTSAYVASFAFARDRLCVPR	Trypsin	3	2724.424	2724.417	Mascot
	28	EAGDACFAKYSYLKNPGEAGDSQEK	Trypsin	3	2734.216	2734.218	Mascot
	29	LCVPRDMSQAGVEYAGNLDYIINSLC	Trypsin	3	3044.407	3044.404	Mascot
	30	S*VITTTISAADAAGRFPSSSDLESVQGNIQR	Trypsin	4	3177.58	3177.59	Mascot
	31	KSVITTTISAADAAGRFPSSSDLESVQGNIQR	Trypsin	3	3305.695	3305.685	Mascot
	32	DRLCVPRDMSAQAGVEYAGNLDYIINSLC	Trypsin	3	3315.529	3315.532	Mascot
	33	D*VDHYMRLVNYCLVVGGTGPVDEWGIAGAR	Trypsin	4	3319.586	3318.591	Mascot
	34	MKSVITTTISAADAAGRFPSSSDLESVQGNIQR	Trypsin	4	3436.702	3436.726	Mascot

Amino acid sequence analysis of R-phycoerythrin alpha subunit. The data show the sequences derived after trypsin digestion. * denotes oxidized methionine.

**Table 2:** Mascot search results.

Accessions	Description	Organism	Prot_Score	MW	PI	Coverage
D6QTA5	Phycoerythrin alpha subunit	*Pyropia yezoensis*	30,804	17,972	5.40	100.0
D6QTA4	Phycoerythrin beta subunit	*Pyropia yezoensis*	24,573	18,810	6.23	77.4
Q1XDB0	C-phycocyanin beta chain	*Pyropia yezoensis*	3196	18,360	4.94	84.9
M4QTP4	Allophycocyanin alpha subunit	*Pyropia yezoensis*	179	17,669	5.06	49.7
Q1XDA9	C-phycocyanin alpha chain	*Pyropia yezoensis*	82	17,567	7.71	24.1
M4QTY0	Ribosomal protein L12	*Pyropia yezoensis*	46	13,752	4.61	15.5

The Mascot results showed 100% similarity with the R-phycoerythrin alpha subunit isolated from *P. yezoensis*. The protein sequence data were reported in the UniProt Knowledgebase (http://www.uniprot.org/uniprot, last accessed on 23 October 2021) under accession numbers.

## Data Availability

The data presented in this study are available from the authors.

## Sample Availability

The R-phycoerythrin alpha subunit extracted from *Pyropia yezoensis* are available from the authors.
